# Spatiotemporal Brain Transcriptomics Reveal Risk Gene Hot-Spots in Major Neuropsychiatric Disorders

**DOI:** 10.21203/rs.3.rs-7667905/v1

**Published:** 2025-10-08

**Authors:** Weiqing LIU, Tomomi Shimogori

**Affiliations:** Shanghai Pudong New Area Mental Health Center, School of Medicine, Tongji University; RIKEN Center for Brain Science

## Abstract

The temporal onset of polygenic brain disorders has been closely linked to the developmental dynamics of genome-wide risk gene expression. In this study, we systematically characterized the spatiotemporal expression patterns of these risk genes and their relevance in differentiating major neuropsychiatric disorders. We analyzed genome-wide risk gene sets for Intelligence Quotient (IQ), Autism Spectrum Disorders (ASD), Attention Deficit Hyperactive Disorder (ADHD), Tourette’s Syndrome (TS), Obsessive Compulsive Disorder (OCD), Anorexia Nervosa (ANO), Neuroticism, Panic disorder, Major Depressive Disorder (MDD), Bipolar Disorder (BIP), Schizophrenia (SZ), Epilepsy, Alzheimer’s Disease (AD), and Parkinson’s Disease (PD). Our results reveal distinct patterns of spatiotemporal enrichment across these traits, allowing their classification into three clusters. To validate the biological significance of these enrichment patterns, we integrated clinical MRI datasets and confirmed structural alterations within the identified spatiotemporal “hot-spots”. Furthermore, by combining gene co-expression network analysis and single-cell transcriptomic data, we delineated the cell-type specificity and functional pathways underlying risk gene enrichment. *In situ* hybridization data from the marmoset brain further provided a comprehensive map of risk gene related module expression. This work reinforces the link between dynamic gene expression and disease mechanisms, while highlighting potential biomarkers and therapeutic targets arising from these identified “hot-spots” and pathways.

## Introduction

Neuropsychiatric disorders frequently manifest during specific developmental windows, suggesting a crucial role for the timing of gene expression in the pathogenesis of these conditions ^1,2^. Advances in genomics have facilitated the identification of extensive genome-wide risk gene sets associated with various psychiatric and neurologic disorders ^3–10^. These risk genes, however, often exert minor to median effects individually, contributing to disease mechanisms in an additive or interactive manner ^11^. The complexity of these disorders underscores the importance of understanding how the spatiotemporal dynamics of risk gene sets expression contribute to disease mechanisms and clinical characteristics.

A comprehensive analysis of the spatiotemporal dynamics of the transcriptome could reveal the anatomical and developmental patterns underlying brain maturation and regional specification, providing deeper insights into their intrinsic molecular mechanisms, and indicating potential “hot-spots” for neuropsychiatric risks operates ^1,12^. Previous studies have highlighted the correlation between the temporal expression patterns of risk genes and the age of disease onset for polygenic neuropsychiatric disorders ^13^. However, the extensive spatiotemporal pattern of risk gene expression—where and which cell types in the brain these genes are co-expressed during development—and its role in distinguishing between different neuropsychiatric disorders remain less explored.

This study aims to bridge this knowledge gap by investigating the spatiotemporal expression patterns of the whole risk gene set derived from unbiased genome-wide studies in such neuropsychiatric disorders as IQ, ASD, ADHD, TS, OCD, ANO, Neuroticism, Panic disorder, MDD, BIP, SZ, AD, PD, and Epilepsy. We hypothesize that these patterns not only correlate with the clinical characteristics but also are useful in differentiating various neuropsychiatric disorders. By analyzing the risk gene set expression enrichment (overrepresentation of risk gene sets relative to the left non-risk genes in the transcriptome) in different brain regions and cell types, we seek to identify neuronal “hot-spots” across major brain regions and throughout the development that may indicate heightened susceptibility to these disorders.

Furthermore, the spatiotemporal co-expression modules identified in this study may reveal novel pathogenic pathways, providing a more nuanced understanding of how risk genes contribute to the development and differentiation of neuropsychiatric disorders. To validate our findings, we utilize clinical MRI datasets, offering an additional layer of evidence that supports the spatiotemporal gene expression patterns observed. By integrating genomic data with clinical imaging, we aim to advance our understanding of the pathogenesis of psychiatric disorders and identify potential biomarkers for disease differentiation and early detection.

This integrated approach not only enhances our understanding of the brain transcriptome’s spatiotemporal dynamics but also sheds light on the intricate relationships between neurodevelopment and the pathogenesis of certain brain disorders.

## Results

### Research schema

**Figure 1:**
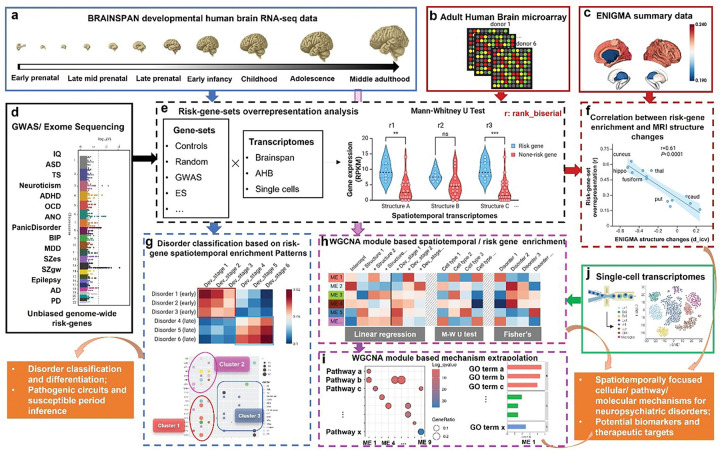
Schematic overview of the analysis workflow. To characterize the spatiotemporal expression and enrichment patterns of risk gene sets associated with 15 major neuropsychiatric traits, we integrated multiple transcriptomic datasets and analytical approaches. a, The BrainSpan developmental RNA-seq dataset was used to assess gene set expression dynamics across brain regions and developmental stages. b, AHBA microarray data complemented BrainSpan data with spatially resolved adult brain expression profiles. c, ENIGMA neuroimaging summary statistics were incorporated to examine correlations between gene expression and brain structural alterations. d, Curated landmark risk-gene databases provided trait-specific gene sets for analysis. e, Gene set overrepresentation analyses identified temporally and regionally enriched expression patterns across traits. f, Correlation analysis linked risk gene expression to ENIGMA-derived brain structural changes. g, Spatiotemporal enrichment profiles revealed convergence between risk gene overrepresentation and trait-specific peak age of onset and pathogenic circuits, allowing clustering of the 15 traits into three distinct groups. h, WGCNA-based linear regression and enrichment analysis identified modules of co-expressed genes linked to disease-relevant brain regions, cell types and developmental windows. i, Functional enrichment analyses of module genes highlighted disorder-specific biological processes and candidate therapeutic targets.

The study employed a multi-layered bioinformatic approach to explore the neurodevelopmental mechanisms and putative therapeutic targets of polygenic neuropsychiatric disorders (Fig. 1). Landmark Genome-Wide Association Studies (GWAS), Exome Sequencing (ES) and alike hypothesis-free cardinal risk gene databases were used to identify polygenic risk genes associated with the neuropsychiatric traits (Table 1). RNA-seq datasets from the BrainSpan developmental human brain project, Allen Human Brain Atlas (AHBA) microarray data, and single-cell RNA-seq data were integrated into the analysis pipeline. Gene-set expression enrichment analysis across spatiotemporal transcriptomes identified key patterns of risk gene expression for different neuropsychiatric disorders. The spatiotemporal expression patterns of risk genes linked these genes to disease-relevant brain regions, neural circuits, ages of onset, and diagnostic categories. To further validate these associations, we leveraged summary statistics from the Enhancing Neuro Imaging Genetics through Meta Analysis (ENIGMA) consortium for major neuropsychiatric disorders, which supported the correlations between risk gene set expression enrichment and disorder-related structural brain alterations. Finally, Weighted Gene Co-expression Network Analysis (WGCNA)-based regression model revealed when, where, and in which cell types, the co-expressed gene modules were upregulated and enriched with neuropsychiatric risk genes. In parallel, the module gene enriched Gene Ontology (GO) terms suggests putatively novel pathogenic molecular mechanisms, culminating in the identification of potential spatiotemporal specific molecular mechanisms, biomarkers, and therapeutic targets.

### Putatively early and late expression enrichment control gene sets showed as expected spatiotemporal enrichment patterns

**Figure 2:**
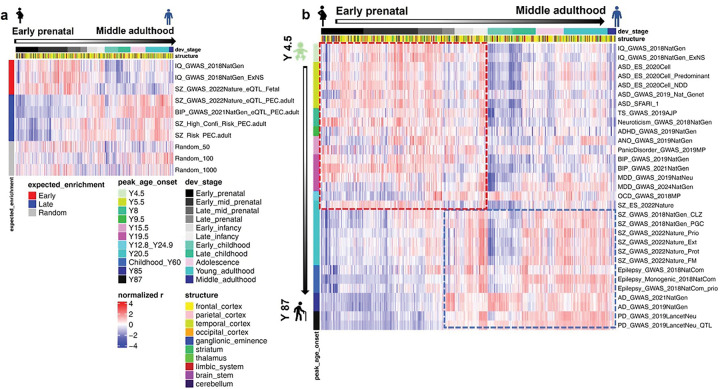
Developmental trajectories of risk gene expression enrichment align with expected patterns and the peak age of onset across 15 neuropsychiatric traits. (a) Putative early and late expression enrichment control gene sets showed as expected spatiotemporal expression enrichment patterns. Gene set expression enrichment analysis of risk gene datasets from IQ GWAS and eQTL-associated genes identified from fetal brain tissues exhibited prominent expression enrichment during prenatal development (early enriched). In contrast, risk gene sets derived from eQTL-associated genes identified from adult brain samples and the PsychENCODE Phase 1 integrative analysis—largely based on adult brain transcriptomes—displayed peak enrichment during adulthood (Postnatal enriched). By comparison, as negative control, randomly generated gene sets showed no consistent spatial or temporal pattern of expression enrichment. Red and blue color gradients represent Z-scored rank biserial correlation coefficients from the Mann–Whitney U test, indicating relative enrichment or deprivation of risk gene sets expression in each sample. (b) Risk gene sets displayed distinct spatiotemporal expression dynamics that corresponded to the typical age of onset for each disorder. Traits (rows) were ordered by increasing peak age of onset, and BRAINSPAN samples (columns) were arranged by ascending developmental age. A clear trend was observed: disorders with later onset exhibited delayed developmental enrichment of associated risk genes, suggesting cumulative polygenic contributions emerging over time.

As shown in Fig. 2a, gene set expression enrichment analysis revealed that risk genes associated with IQ, as well as those with anticipated enrichment in fetal brain tissue, exhibited prominent expression enrichment during prenatal development. In contrast, risk gene sets derived from the PsychENCODE Phase 1 integrative analysis—largely based on adult brain transcriptomes—displayed peak enrichment during adulthood. By comparison, randomly generated gene sets showed no consistent spatial or temporal pattern of expression enrichment. These control analyses collectively support the validity of our approach in capturing disease-relevant spatiotemporal enrichment signatures from gene expression data.

### Developmental expression enrichment patterns of risk gene sets align with peak age of onset across major neuropsychiatric disorders

As shown in Fig. 2b, the genome-wide unbiased risk gene sets exhibited progressively delayed expression enrichment during brain development—from early prenatal stages to middle adulthood—in association with increasing peak ages of disease onset ^14^. This trend suggests an additive or interactive polygenic contribution to the pathogenesis of these disorders and traits. Consistent with previous studies ^13^, most early onset psychiatric disorders demonstrated relatively early (i.e., pre-childhood) developmental enrichment of risk gene expression compared to neurological disorders, whose risk genes were predominantly enriched from late prenatal stages into adulthood (Fig. 2b, Fig. S1).

Notably, the schizophrenia risk gene sets derived from exome sequencing (SZes) and genome-wide association studies (SZgw) displayed divergent developmental trajectories (Fig. 2b, Fig. S1). Specifically, SZgw showed predominant postnatal enrichment, while SZes showed relative enrichment in prenatal to early infancy stages (before 1 year of age), consistent with evidence that rare exonic variants in SZ are more strongly associated with early neurodevelopmental disruptions ^15^.

### Spatial enrichment patterns of neuropsychiatric risk gene sets implicate disease-relevant brain regions and circuits

**Figure 3:**
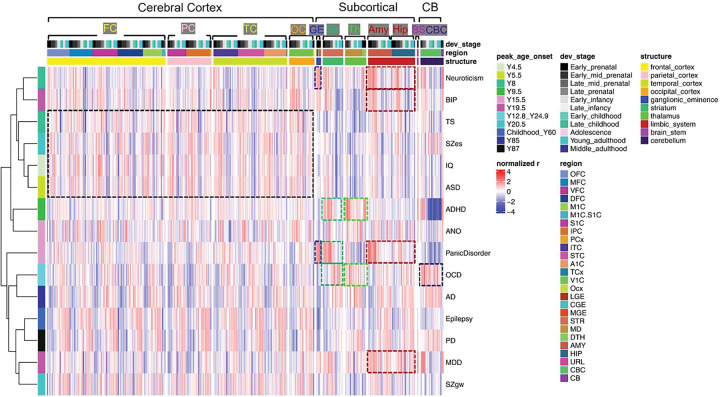
Spatial enrichment patterns of neuropsychiatric risk gene sets implicate disease-relevant brain regions and circuits. BRAINSPAN brain regions were ordered from anterior to posterior and from cortical to subcortical structures. Red and blue color gradients represent Z-scored rank biserial correlation coefficients, indicating relative enrichment or deprivation of risk gene sets expression in each sample. Putative hot-spot regions were identified based on apparent expression enrichment patterns observed in the heatmap, from which Hierarchical clustering separated the 15 traits into 2 clusters, corresponding to that in Fig S1b. The qualitatively evaluated “hot-spots” for each trait were labeled with rectangles with the corresponding color as that in the structure color legend. Across the 15 traits examined, risk gene sets exhibited consistent enrichment across cortical subregions, while subcortical structures showed disorder-specific enrichment profiles, especially for OCD, ADHD, Panic disorder, and mood-related traits, including Neuroticism, BIP, and MDD. In contrast, risk gene sets for IQ and ASD did not show prominent subcortical expression enrichment. For the risk genes of neurological disorders and SZ, seems to be widely expressed across the cortical and subcortical regions.

To make intra-disorder comparisons, we merged the risk gene sets identified by different gene-mapping approaches for each trait (except for SZ, due to the significant disease heterogeneity). Across all the 15 traits, risk gene sets exhibited consistent enrichment patterns within cortical sub-regions. In contrast, subcortical regions demonstrated disorder-specific enrichment profiles (Fig. 3).

Notably, ADHD risk genes showed prominent prenatal expression enrichment in the thalamus (Th) and striatum (Str)-regions implicated in sensorimotor gating and attentional control ^16,17^. Similarly, risk genes for Panic disorder were strongly enriched in the ganglionic eminence (GE), Str, and amygdala (Amy) during prenatal development. These findings align with the well-established roles of Th in attention, and Amy, Str and GABAergic interneurons in emotional processing and arousal ^17–19^. The results support prevailing neurobiological theories implicating thalamic dysfunction in ADHD and amygdala and GABAergic involvement in Panic disorder ^19,20^. Moreover, the Str, increasingly recognized as a key node in anxiety-related circuits ^21,22^, potentially implicating a common disruption in valence processing mechanisms maturation across ADHD and Panic disorder ^23,24^.

For OCD, risk gene sets were significantly overrepresented in the striatum, thalamus, and cerebellum–consistent with the cortico-striato-thalamo-cortical (CSTC) circuitry model ^25^. Recent neuroimaging studies have highlighted abnormal microstructural and functional connectivity between the cerebellum and CSTC circuits in OCD ^26^, further corroborating our findings.

Additional enrichment “hot-spots” were observed in the limbic system (amygdala and hippocampus) for neuroticism, BIP, and MDD. These regions are central to emotion processing and have been consistently implicated in the pathophysiology of these disorders ^27,28^. In contrast, risk gene sets for IQ and ASD did not show prominent subcortical expression enrichment. The risk genes associated with neurological disorders and schizophrenia appeared to be broadly expressed across both cortical and subcortical regions, suggesting that these traits may involve more widespread or diffuse neuropathological processes.

### Spatiotemporal expression patterns of risk gene sets differentiate neuropsychiatric disorder subgroups

To compare the spatiotemporal expression profiles of risk gene sets across disorders and gene-mapping strategies, we scaled the correlation coefficients of all spatiotemporal BrainSpan samples and performed hierarchical clustering. As expected, risk gene sets derived from different datasets or mapping strategies for the same disorder generally clustered closely (Fig. S1a). This analysis also revealed a clear separation between risk gene sets predominantly expressed early versus those expressed later in development (Fig. S1a). Notably, the early-expression cluster was enriched for psychiatric disorders and neurodevelopmental related traits (e.g., IQ, neuroticism), whereas the late-expression cluster was primarily composed of neurological disorders and two psychiatric conditions with bimodal onset ages or neurodegenerative features (e.g., OCD, schizophrenia) ^29,30^.

The 15 merged gene sets yielded clustering patterns comparable to those observed using individual gene sets (Fig. S1b), suggesting that even subset of risk genes derived from relatively small GWAS studies capture meaningful spatiotemporal signatures of disease pathogenesis.

The smoothed developmental dynamics of the 15 traits revealed divergent developmental trajectories between neurological and psychiatric disorders, with OCD exhibiting a pattern more akin to neurological conditions (Fig. S1b and c). Furthermore, both MDD and SZgw showed bimodal risk gene expression peaks (perinatal and adolescence/young adulthood), potentially reflecting two sensitive developmental windows during which environmental insults may exert their effects (Fig. S1b and c).

**Figure 4:**
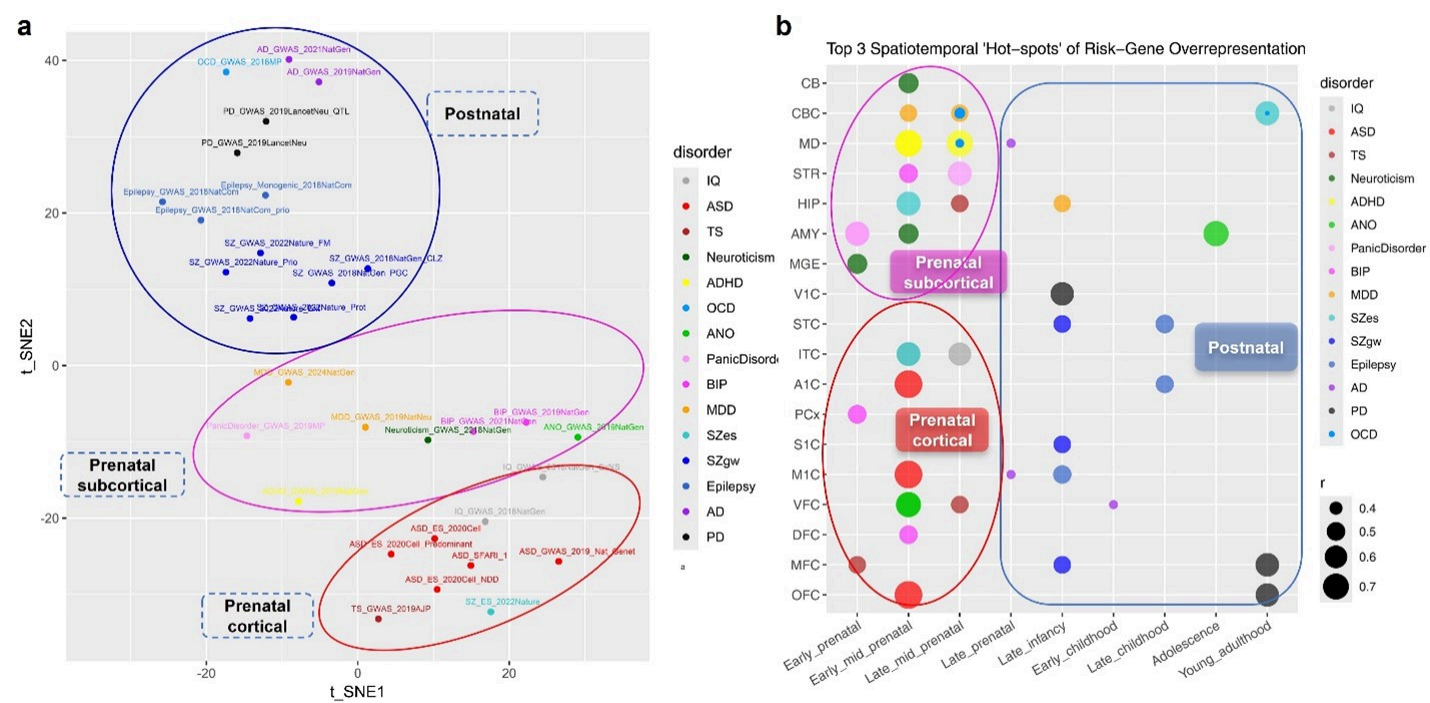
Spatiotemporal expression patterns of risk gene sets differentiate neuropsychiatric disorder subgroups (a) Spatiotemporal expression enrichment profiles of 31 risk gene sets across 524 BrainSpan samples were analyzed using t-distributed Stochastic Neighbor Embedding (t-SNE). The analysis revealed three distinct clusters: (1) an early expressed “Prenatal cortical” cluster comprising IQ, ASD, TS, and SZes; (2) an early expressed “Prenatal subcortical” cluster including BIP, MDD, neuroticism, ANO, and Panic disorder; and (3) a late expressed “Postnatal” cluster encompassing SZgw, OCD, epilepsy, AD, and PD. Cluster identities were defined in reference to prior results shown in Fig. S1. (b) The top three brain region × developmental stage combinations with the highest average r values for each of the 15 merged gene sets are visualized in a two-dimensional matrix, representing trait-specific enrichment “hot-spots”. Bubble sizes represent the average r values across all samples from the corresponding region-stage combination. A similar tripartite organization was observed, corresponding to the “Prenatal cortical” (red ellipse), “Prenatal subcortical” (pink ellipse), and “Postnatal” (blue square) clusters identified in the t-SNE analysis. These spatiotemporal enrichment patterns broadly align with known disorder-related circuit mechanisms. Notably, although the ADHD risk gene set showed spatial enrichment in the mediodorsal thalamus, it was positioned near the “Prenatal cortical” cluster, possibly reflecting a spatiotemporal pattern overlapped with ASD.

To further delineate the spatiotemporal dimensional relationships among major neuropsychiatric disorders, we performed a t-distributed stochastic neighbor embedding (t-SNE) analysis using the r values representing spatiotemporal enrichment of risk gene expression. As shown in Fig. 4a, the disorders and associated risk gene sets could be broadly classified into three distinct clusters. Integrating the prior analyses of spatiotemporal enrichment patterns (Figs. 2 and 3), we infer that the first t-SNE dimension (tSNE1) primarily captures temporal variation, while the second (tSNE2) reflects spatial differences. Based on their relative positions in the t-SNE space, we defined three major categories: (1) a “Prenatal cortical” cluster comprising IQ, ASD, TS, and SZes (Fig. 3, Fig. 4a); (2) a “Prenatal subcortical” cluster including BIP, MDD, neuroticism, ANO, and Panic disorder (Fig. 4a); and (3) a “Postnatal” cluster encompassing SZgw, OCD, epilepsy, AD, and PD (Fig. S1a, Fig. 4a).

To further visualize the spatiotemporal enrichment for each of the 15 merged risk gene sets, we mapped the top three “hot-spots” (i.e., developmental stage and brain region combinations with the highest average r values) across major structures in BrainSpan (Fig. 4b). These enrichment hot-spots broadly mirrored the t-SNE-based clustering and aligned with known disorder-related pathological mechanisms. Furthermore, the expression enrichment of risk genes for multiple “Prenatal cortical” and “Prenatal subcortical” traits during the mid-prenatal stages coincides with earlier studies, which found a substantial expression peak for pleiotropic risk genes of multiple disorders ^31^, implicating the involvement of such neural developmental processes in this period as neural differentiation and migration in the pathogenesis of those psychiatric disorders. For instance, the medial dorsal thalamus (MD) at mid-prenatal was among the top-enriched hot-spots for ADHD and OCD, while the Amy at early-prenatal was most prominent for Panic disorder. Additionally, the enrichment of ANO risk gene expression in Amy and VFC coincides with the abnormal functional connectivity in the corticolimbic circuitry in this disorder ^32^. Interestingly, inferior temporal cortex (ITC) and hippocampus (HIP) at mid-prenatal and cerebellum cortex (CBC) at adulthood were among the top 3 hot-spots for SZes, while that for SZgw were mapped to medial frontal-primary sensory-superior temporal cortex at late infancy, implicating divergent pathogenic mechanisms for different SZ subtypes.

### Spatiotemporal enrichment patterns but not the shared risk genes drive the classification of the traits

To exclude the possibility that trait clustering was driven by shared risk genes across datasets, we quantified gene set overlap among the 15 traits using pairwise Jaccard similarity test. As shown in Fig. S2a-c, traits within the same t-SNE clusters-such as SZes, IQ, and TS in the “Prenatal cortical” cluster, and ADHD and Panic disorder in the “Prenatal subcortical” cluster—exhibited minimal gene overlap. Overall, pairwise Jaccard similarities were low, with the highest (J = 0.15) observed between MDD and neuroticism (Fig. S2c, d). Other traits within the same spatiotemporal clusters, including SZgw, AD, PD, and epilepsy in “Postnatal”, as well as IQ, ASD, and TS in “Prenatal cortical”, showed generally lower similarities with each other than that with traits from different clusters (Fig. S2c, d), reinforcing the notion that clustering reflects convergent expression dynamics rather than shared genetic architecture.

### Assessing the biological relevance of risk gene expression patterns using ENIGMA brain morphometry

**Figure 5:**
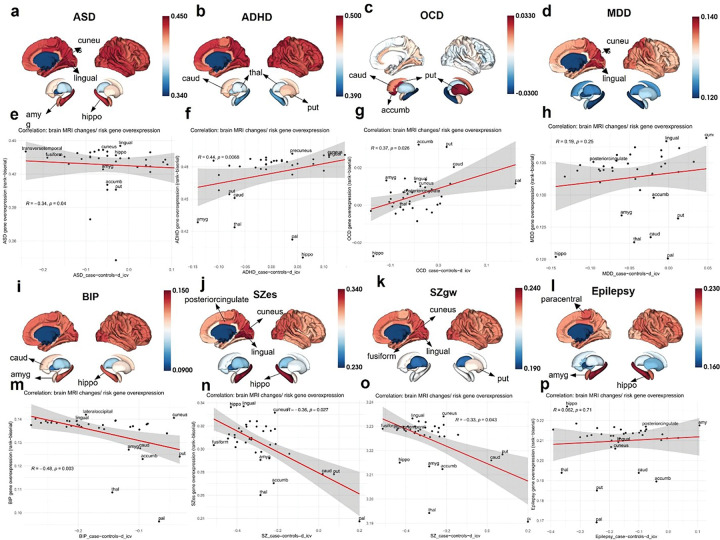
Spatial enrichment of risk gene sets in ENIGMA brain regions and their association with MRI-derived structural alterations in major psychiatric disorders. Thirty-eight brain regions shared between the AHBA microarray dataset and ENIGMA Toolbox anatomical structures were manually extracted and aligned for correlation analyses. For each disorder: (a) ASD, (b) ADHD, (c) OCD, (d) MDD, (i) BIP, (j) SZes, (k) SZgw, and (l) epilepsy, regional enrichment patterns of risk gene expression were visualized by projecting the rank-biserial correlation coefficients values onto a FreeSurfer brain template. Warmer colors indicate stronger enrichment of risk gene sets. Regional enrichment “hot-spots” are labeled accordingly. Risk genes for ASD, ADHD, and BIP exhibited widespread cortical expression enrichment, while those for SZ and MDD showed predominant enrichment in the occipital cortex—particularly in the lingual and cuneus regions. Subcortical structures displayed trait-specific enrichment patterns—for example, the striatum was strongly enriched with OCD risk genes, and the hippocampus with risk genes for ASD, BIP, SZ, and epilepsy. Spearman correlation analysis was conducted between regional risk gene enrichment and structural brain alterations (indexed by d_icv) from ENIGMA meta-analyses. The red line represents the fitted linear model, and the shaded area indicates the 95% confidence interval of the fit. Negative correlations were observed for (e) ASD (*R*= −0.34, *P* =0.04), (m) BIP (*R*= −0.48, *P* =0.003), (n) SZes (*R*= −0.36, *P* = 0.027), and (o) SZgw (*R*= −0.33, *P* = 0.043), indicating that higher regional enrichment of risk genes was associated with greater volume reductions in those regions. In contrast, positive correlations were detected for (f) ADHD (*R*= 0.44, *P* = 0.0068) and (g) OCD (*R*= 0.37, *P* = 0.026). No significant associations were found for (h) MDD and (p) epilepsy (*P* >0.05).

To further assess the biological relevance of the spatiotemporal expression enrichment patterns of risk gene sets, we examined their associations with meta-analyzed structural brain alterations from the ENIGMA consortium. A total of 231 anatomically defined brain regions were extracted from six adult human donors in the Allen Human Brain Atlas (AHBA) microarray dataset (Fig. S3). Of these, 38 regions overlapped with the ENIGMA summary data and were retained for visualization and correlation analyses. The enrichment patterns of risk gene expression for eight available ENIGMA neuropsychiatric disorders were projected onto a FreeSurfer brain template (Fig. 5a–d, i–I). Notably, risk genes for ASD, ADHD, and BIP showed widespread expression enrichment across the neocortex. In comparison, SZ and MDD risk genes exhibited relatively stronger enrichment in the occipital cortex—particularly in the lingual and cuneus regions. Furthermore, subcortical structures showed marked disorder-specific patterns. For instance, the striatum is significantly enriched with OCD risk genes, while hippocampus is enriched with ASD, BIP, SZes and Epilepsy risk genes.

Intriguingly, significant negative correlations were observed between MRI-derived structural alterations and regional expression enrichment of risk gene for ASD, BIP, and SZ (Fig. 5e, m–o), whereas positive correlations emerged for ADHD and OCD (Fig. 5f and g). The former may reflect accumulated dampen effects of risk genes on brain development in these disorders characterized by “loss-of-functions”, while the latter may reflect enhanced neural circuit activity associated with heightened functional phenotypes such as hyperactivity in ADHD and compulsivity in OCD. In contrast, no significant associations were detected for MDD and epilepsy (Fig. 5h and p), suggesting that risk gene enrichment in these disorders may exert more “functional” rather than structural effects across the brain regions examined. Together, these findings highlight that spatial expression “hot-spots” of risk genes may serve as potentially modifiable targets for therapeutic intervention across neuropsychiatric conditions.

### WGCNA-derived co-expression modules and their functional implications in neuropsychiatric disorders

We applied Weighted Gene Co-expression Network Analysis (WGCNA) to the BrainSpan transcriptomic dataset to identify modules of co-expressed genes across developmental stages and brain regions. A total of 41 co-expression modules were initially identified (Fig. S4), of which 32 modules exhibited significant spatial or temporal expression enrichment (defined as at least one dummy variable with a B value > 0.05 in the linear regression models) and were retained for downstream analysis (Fig. S5a). Notably, modules with distinct spatiotemporal expression profiles exhibited different enrichments in different neuropsychiatric disorders (Fig. S5b). Even after rigorous Bonferroni correction, four modules show significant enrichment in six disorders (Fig. S5c). Furthermore, the expression enrichment of module genes in each cell type were evaluated to further infer the enrichment of the spatiotemporal modules at cell type levels. As shown in Fig. S6, modules overrepresented during early developmental stages were enriched in fetal cell types relative to adult cells. In contrast, modules predominant in the perinatal and early childhood stages showed enrichment in glial cells, suggesting the roles of functional specialization for these module genes in glia development trajectories, synaptogenesis and pruning, myelinization, and maturation of the blood brain barrier ^33,34^. Notably, genes in the module “MEdarkgreen”, which were overexpressed in the fetal neocortex according to regression analysis, also showed selective enrichment in fetal quiescent cells and adult neocortical neurons. This pattern suggests an early developmental specification of adult neocortical neurons ^35^.

**Figure 6:**
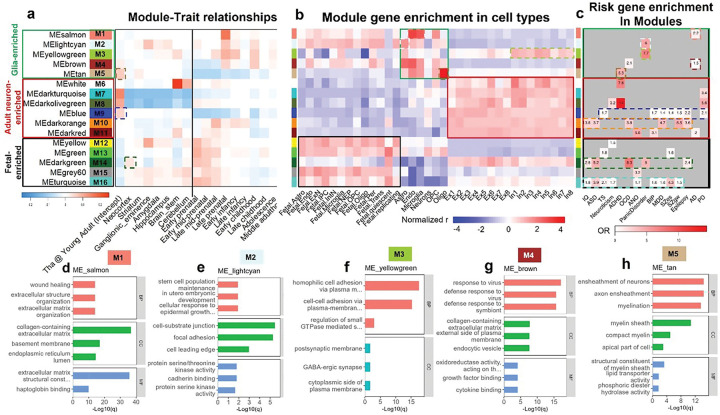
Spatiotemporal co-expression module-based risk gene enrichment and extrapolated pathogenic mechanisms. To further prioritize biologically relevant modules, we focused on the 16 modules that showed the strongest enrichment for trait-associated risk genes. Specifically, (c) the top three modules per trait were selected based on odds ratios, which were. These were then integrated with (b) single-cell transcriptomic enrichment profiles to generate a composite map that links the spatial, temporal, and cellular dimensions of disease vulnerability. The color gradients represent Z-scored rank biserial correlation coefficients from the Mann-Whitney U test. Notably, (a) the top enriched modules could be broadly categorized into three major groups: Glia-enriched, Adult-neuron-enriched, and Fetal-enriched modules, as demarcated by different rectangles in panels a-c. Note that, two modules each in Adult-neuron-enriched (M9 and M10) and Fetal-enriched groups (M14 and M16) were significantly associated with multiple neuropsychiatric disorder risks. (d–h) Functional annotation of the Glia-enriched modules—M1–5—highlighted distinct Biological Processes (BP), Cellular Components (CC), and Molecular Functions (MF) implicated in neuropsychiatric disorders. The top three Gene Ontology (GO) terms in each category are shown; significance was determined using a q-value threshold of 0.05. By jointly analyzing their spatiotemporal expression patterns, cell-type specificity, and risk gene enrichment, we extrapolated key pathogenic hot-spots across multiple scales and inferred molecular mechanisms disrupted *in situ*.

To further prioritize biologically relevant modules, we focused on 16 that exhibited the strongest enrichment for disorder-associated risk genes by selecting the top three modules per trait based on odds ratios (Fig. 6c). These modules were then integrated with single-cell transcriptomic enrichment profile to construct a composite map linking the spatial, temporal, and cellular dimensions of disease vulnerability (Fig. 6a–c). Notably, the top risk gene-enriched modules could be broadly categorized into three groups: Glia-enriched, Adult-neuron-enriched, and Fetal-enriched modules (Fig. 6a and b). The divergent cell type expression enrichment patterns between the Glia-enriched and Adult-neuron-enriched modules were replicated with another single-cell transcriptome dataset derived from the adult brain (Fig. S7).

Among the Glia-enriched group, M1 and M4 were significantly overrepresented in endothelial cells (Endo), microglia, astrocytes, and oligodendrocyte progenitor cells (OPCs) during the late prenatal and early childhood periods (Fig. 6a and b). Both modules showed strong associations with AD risk (Fig. 6c). GO analyses revealed that M1 and M4 were enriched for functions such as Extracellular Matrix (ECM) organization and immune response (Fig. 6d and g), aligning with recent findings of dysregulated Endo, astrocytic, and oligodendrocytic functions in the AD prefrontal cortex ^36^. Similarly, M2 and M3—two other Glia-enriched modules expressed during late prenatal and early childhood stages—were functionally linked to cell adhesion, migration, and GABAergic synapse formation (Fig. 6e and f). These modules were nominally associated with Panic disorder risk, supporting the hypothesis that abnormal migration and functional integration of GABAergic interneurons contribute to Panic disorder pathophysiology ^37,38^. In contrast, M5, an oligodendrocyte significantly enriched module showing peak expression in the adult thalamus and striatum and functionally related to axon myelination (Fig. 6h), was marginally associated with ADHD genetic risk. This finding lends support to the hypothesis that myelination deficits and white matter abnormalities may underlie the pathogenesis and persistence of ADHD ^39,40^.

**Figure 7:**
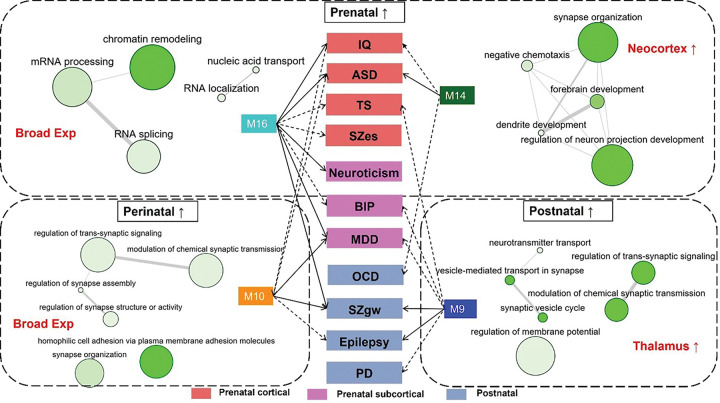
Pleiotropic and polygenic effects of co-expression modules across major neuropsychiatric disorders. Four co-expression modules exhibited pleiotropic and polygenic associations with multiple major neuropsychiatric disorders. Module M16, broadly overrepresented during early prenatal development, was significantly associated with multiple traits, including “Prenatal cortical” traits (IQ, ASD, TS, and nominally SZes), “Prenatal subcortical” traits (Neuroticism, MDD, and nominally BIP), as well as “Postnatal” SZ genome-wide association (SZgw). Functional annotation of M16 revealed enrichment in chromatin organization and epigenetic regulation, pointing to a common early developmental origin underlying these disorders. In contrast, M14 was preferentially expressed in the neocortex during early prenatal development and was significantly associated with ASD, and nominally with IQ and OCD. Enrichment in “forebrain development” and “synapse organization” pathways suggest spatiotemporally specific mechanisms contributing to these phenotypes. M10, a module enriched in perinatal and adult neurons, was functionally related to synaptic organization and signaling. It was significantly associated with MDD and SZgw, and nominally with IQ, ASD, and Epilepsy, suggesting the involvement of synaptic formation and stabilization in the pathophysiology of these disorders. Finally, M9, which showed significant association with postnatal SZgw and Epilepsy, and nominal associations with PD, MDD, BIP, and TS, was enriched in adult thalamic neurons. Its associated gene functions related to synaptic signaling imply a potential deficit in information relay and integration in these conditions. Semantic similarity-based visualizations of pleiotropic Gene Ontology (GO) biological processes were generated using REVIGO. In the resulting bubble plots, the color intensity of each bubble represents the −log_10_-transformed P-value (darker indicates greater significance), while bubble size reflects the frequency of the GO term in the UniProt database, with larger bubbles corresponding to broader, more general terms and smaller bubbles to more specific ones. Lines between bubbles represent semantic similarity between GO terms, with thicker lines indicating stronger similarity.

As for the Adult-neuron-enriched and Fetal-enriched co-expression modules, both showed significant associations with multiple neuropsychiatric disorders, particularly M9–10, M14, and M16 (Fig. 6a–c, Fig. 7). Among the modules, M14 and M16 showed pronounced expression during early developmental stages across multiple cortical and subcortical regions (Fig. 6a and c). Notably, M14 exhibited specific enrichment in quiescent cells of the fetal neocortex and maintained relative expression enrichment in adult excitatory neurons (Ex) (Fig. 6b). GO terms associated with M14, including “forebrain development” and “synapse organization” (Fig. 7, Fig. S9), highlight a shared developmental origin and functional convergence underlying prefrontal cortex deficits observed in intellectual disability, ASD, and OCD. In comparison, M16, which shows early expression in the ganglionic eminence (GE) and cerebellum, functionally related with chromatin structure and epigenetic modifications (Fig. 6a–c, Fig. S9, Fig. S10), may represent a common early fetal development deficit among the pathogenesis of multiple psychiatric disorders (Fig. 6c, Fig. 7). In contrast, M12, specifically enriched for neuroticism-associated risk genes, peaked in expression during the earliest developmental phases within the GE, suggesting that temperamental traits may be rooted in stem cell proliferation and migration processes in the GE (Fig. 6a–c, Fig. S9).

As expected, several co-expression modules enriched in adult neurons exhibited significant correlations with both psychiatric and neurological disorders. Notably, many of these modules were spatially enriched in discrete brain regions—such as the thalamus and brainstem—supporting the notion that postnatal functional specification of these hot-spot structures may contribute to the pathogenesis of specific neuropsychiatric conditions (Fig. 6a–c). M9, which is enriched in adult thalamic neurons, showed significant associations with SZgw and Epilepsy, and nominal associations with PD, MDD, BIP, and TS. The gene functions within M9 were primarily related to synaptic signaling, suggesting potential deficits in information relay and integration in these disorders. By contrast, another pleiotropic module, M10, which is enriched during perinatal stages, lacked significant spatial enrichment but was functionally linked to synaptic organization and signaling. M10 showed significant associations with MDD and SZgw, and nominal associations with IQ, ASD, and Epilepsy, indicating a potential role of synaptic formation and stabilization in the pathophysiology of these conditions.

Intriguingly, PD risk genes were preferentially enriched in thalamic modules M7–9, with elevated expression observed during both early developmental periods and adulthood (Fig. 6a and c), highlighting the potential involvement of thalamic circuits across the lifespan in PD vulnerability. Enriched GO terms for M7–9 includes “Ach receptor”, “peptide receptor”, and “synaptic transmission” (Fig. S8), in line with the thalamus functions and pathophysiology of PD.

We further explored the Kyoto Encyclopedia of Genes and Genomes (KEGG) pathways enriched in these modules. Eleven of the 16 modules show significant (q-value FDR < 0.05) enrichment for KEGG pathways (Fig. S10). with the most significant pathway is “cell cycle”, enriched in M12, which are also enriched for neuroticism risk genes and cell-cycle related GO terms (Fig. S9).

### Exemplar module-specific risk gene expression across the neonatal marmoset brain

Due to the absence of fine-resolution, whole-brain gene expression data in the human neonate, we examined *in situ* hybridization (ISH) data from the neonatal marmoset-a New World primate-to assess the spatial expression of representative genes identified in the co-expressed modules enriched for neuropsychiatric risk.

As shown in Fig. S11, the ADHD-associated gene FOXP2 (from M8) exhibited strongest expression in the thalamus, consistent with its known role in sensorimotor and cognitive integration ^17^. Furthermore, the M5 module gene PLP1, which is overexpressed in both the thalamus and oligodendrocytes, exhibits prominent expression in the white matter of the neonatal marmoset brain and is markedly upregulated at 6 months of age, suggesting its potential involvement in the pathogenesis of ADHD. Additional examples include neuroticism-associated genes PAX6 and KDM3A (both from M12), which exhibited enriched expression in the GE, hippocampus, amygdala, and cerebellum (Fig. S12a and b). Likewise, cortex-enriched M14 genes-SYT6 (OCD-associated) and SATB1 (ASD-associated), were preferentially expressed in the cerebral cortex (Fig. S13a and b). Representative M16 ASD risk gene ADNP and SZes risk gene SRRM2, all exhibit widespread expression across the neonatal marmoset brain, consistent with the spatiotemporal expression profile of M16 (Fig. S14a and b). These high-resolution, whole-brain ISH data from the neonatal marmoset provide independent validation of the disorder-related spatiotemporal “hot-spots” identified through WGCNA and regression analysis. Moreover, the distinct spatial expression profiles of these module/risk genes at cellular resolution provide a comprehensive overview of brain structures implicated in disease pathogenesis, including regions that may have been overlooked in prior studies.

## Discussion

The findings of this study reveal significant insights into the spatiotemporal dynamics of risk gene expression and their implications for understanding neuropsychiatric disorders. Our analysis demonstrates that the spatiotemporal expression patterns of genome-wide risk gene sets are not only reflective of the temporal onset of these disorders but also serve as distinguishing features and pathogenesis extrapolations for different groups of neuropsychiatric conditions.

The spatiotemporal expression enrichment patterns observed across neuropsychiatric disorder risk genes underscore the importance of developmental timing and regional specificity in disease pathogenesis. Consistent with prior studies, our results confirm that traits with earlier peak onset ages, such as IQ, ADHD, and ASD show prenatal and early postnatal expression enrichment of risk genes ^13^. In contrast, late-onset disorders, such as PD and AD, display enriched gene expression during adulthood. The differentiation between early and late-onset disorders based on spatiotemporal gene expression supports the hypothesis that neurodevelopmental and neurodegenerative disorders have distinct transcriptional dynamics.

Notably, the risk gene set spatiotemporal expression enrichment dynamics for certain disorders, such as MDD and SZ, exhibit dual peaks, corresponding to distinct phases of heightened susceptibility. Furthermore, prolonged OCD risk gene expression enrichment from prenatal neocortex to adult striatum aligns with clinical observations of bimodal onset peaks for this disorder, suggesting that different pathogenic pathways may contribute to the disorder at different developmental stages ^41^. These findings highlight the importance of integrating temporal dynamics into neuropsychiatric disorder models, as they may uncover previously overlooked windows of vulnerability and therapeutic opportunity.

Our study identified disorder-specific spatial “hot-spots” for risk gene expression, aligning with known neurobiological mechanisms. For example, ADHD risk genes showed prominent increased expression enrichment in the thalamus and striatum, regions implicated in attention and sensorimotor integration ^16,17^. Similarly, OCD risk genes were enriched in the CSTC circuits, consistent with established models of OCD pathophysiology ^42^. The limbic system emerged as a key site for risk gene expression in mood disorders such as MDD and BIP, supporting its role in emotional processing abnormalities ^28^.

Interestingly, cortical regions showed consistent expression patterns for IQ and ASD, reinforcing the cortical-centric nature of these conditions. Conversely, subcortical regions displayed distinct expression patterns in emotional disorders, such as Panic disorder, OCD, MDD, BIP and neuroticism. Moreover, neurodegenerative disorders and schizophrenia appeared to broadly affect both cortical and subcortical regions, indicating widespread neuropathological processes. These findings suggest that spatial specificity of gene expression may offer critical insights into the underlying neural circuits and their roles in the pathogenesis of specific disorders.

Clustering analyses revealed clear distinctions between neurodevelopmental and neurodegenerative disorders based on their spatiotemporal gene expression patterns. Early-onset psychiatric disorders such as ASD and SZes clustered separately from late-onset conditions, including AD and PD. This separation highlights the utility of spatiotemporal gene expression data in identifying disease trajectories and potential biomarkers for early differentiation.

The observation that different risk gene sets for the same disorder generally clustered closely, and this spatiotemporal similarity could not be explained by shared risk genes supports the robustness of this spatiotemporal expression enrichment pattern-based disorder differentiation and comparison. Moreover, the clustering of risk gene sets for the same disorder such as schizophrenia into both prenatal and postnatal enriched categories suggests the presence of complex, multi-phase pathogenic mechanisms.

The validation of our findings using ENIGMA MRI datasets further underscores the translational potential of spatiotemporal gene expression analysis. Correlations between risk gene expression patterns and structural brain changes suggest that these “hot-spots” could serve as biomarkers for disease detection, monitoring, and therapeutic targeting. For instance, the negative correlation between MRI structural changes and risk gene expression in ASD, BIP, and schizophrenia highlights the potential for targeted interventions aimed at modulating these circuits during critical developmental periods.

Additionally, we explored the expression enrichment patterns of the 15 major neuropsychiatric traits in human brain single-cell RNA-sequencing data to speculate the contribution of different cell types in the pathogenesis of these disorders. As shown in Fig. 6, several disorders show obvious cell-type specific risk gene expression enrichment. Such as AD risk genes were most significantly overrepresented in such glia cells like endothelial cells, astrocytes, and microglia (Fig. 6b), all play vital roles in the pathogenesis of AD. Another typical cell-type specific expression enrichment is Panic disorder risk genes in astrocyte and inhibitory neurons (Fig. 6b, Fig. S3b), in accordance with the significant enrichment of those risk genes in M2, which is upregulated in GE (Fig. 6a), the developmental origin of inhibitory neurons in the brain. On the contrary, ADHD risk-genes are overrepresented in excitatory neurons and oligodendrocytes in the thalamus, in accordance with their spatiotemporal enrichment patterns (Fig. 6, Fig. S3b and Fig. 3). Interestingly, PD risk genes are also significantly enriched in the modules related with thalamus, which has long been found to be related with the pathological changes in Parkinson’s disease ^43^.

Furthermore, WGCNA based GO and KEGG analysis indicate that cell-cycle dysfunction in GE during very early development (early fetal) may play a critical role in the pathogenesis of neuroticism. In comparison, the perinatally overexpressed module, M1, which is most significantly enriched for AD risk genes (Fig. 6c), is also enriched for such pathways related with Complement cascades and ECM-receptor interactions (Fig. S10), both have been proposed to participate in the critical pathogenesis of AD ^44,45^. Intriguingly, the significant perinatal upregulation of these immune related functions may indicate the involvement of early life acquired immune system maturation and microglia activation in later life exaggerated inflammatory brain damage and decreased cognitive reserve seen in AD ^46^. In comparison, M9, which is enriched with risk genes for many neuropsychiatric disorders (TS, BIP, MDD, SZgw, Epilepsy, PD), is specifically upregulated in the adult thalamus (Fig. 6a, c), supporting the reported thalamus abnormalities in those disorders ^43,47–49^. KEGG and GO analysis indicate that synaptic function, such as Adrenergic and Cholinergic neurotransmissions in the thalamus may play important roles in the pathogenesis of information processing, sensory gating, and perception deficits commonly seen in those neuropsychiatric disorders ^48,50–54^. Furthermore, other co-expression modules (M4–5, M7–8), while showing eigengene upregulation in the thalamus, either prenatally or postnatally (Fig. 6a), also enriched with risk genes for multiple neuropsychiatric disorders (ADHD, OCD, AD, PD) that repeatedly found to be related with thalamic dysfunctions ^54–57^. The biological functions of the thalamic prominent co-expression modules, like axon myelination, acetylcholine receptors, neuropeptide signaling and immune related functions, provide novel insights and therapeutic targets for these disorders (Fig. 6–7, Fig. S8).

Another two “pleiotropic” co-expressed modules enriched with risk genes for multiple neuropsychiatric traits are M14 and M16, both overexpressed during early life stages in the cortex and subcortical regions (Fig. 6–7). Function analysis of these 2 modules validated the importance of forebrain synapse organization in cognition development, ASD, and OCD pathogenesis, and the shared mechanism of transcription regulation changes in multiple neuropsychiatric disorders.

Finally, the distinct spatiotemporal expression profiles of the risk gene sets and the enriched biological pathways observed in this study may inform precision medicine approaches. Therapeutic strategies could focus on modulating gene expression or neural activity in specific regions during vulnerable developmental windows, potentially mitigating disease progression.

This study provides a foundation for exploring several promising research avenues. First, further integration of multi-omics data, including epigenomics and proteomics, could enhance our understanding of the molecular mechanisms underlying spatiotemporal gene expression patterns. Second, longitudinal studies linking gene expression dynamics with clinical outcomes could elucidate the causal relationships between risk gene expression and disease progression. Finally, experimental validation of identified “hot-spots” using animal models and advanced neuroimaging techniques could pave the way for novel therapeutic interventions.

In conclusion, our findings demonstrate that spatiotemporal co-expression modules of risk gene set not only correlate with the onset of neuropsychiatric disorders but also provide critical insights into their differentiation and pathogenesis. These results highlight the potential for leveraging spatiotemporal gene expression patterns as biomarkers and therapeutic targets, offering new avenues for advancing the diagnosis and treatment of major psychiatric disorders.

## Materials and methods

### Genome-wide risk genes for the 15 traits

To validate the feasibility of using the spatiotemporal expression patterns of risk gene sets to infer key pathogenic “hot-spots”—and thereby estimate the peak age of onset and the vulnerable brain regions for various neuropsychiatric traits—we employed both positive and negative controls. As positive controls, we selected risk gene sets expected to show stage-specific enrichment, such as IQ risk genes and eQTL-associated genes identified from fetal or adult brain tissues. As negative controls, we used randomly generated gene sets with varying sample sizes.

To avoid bias in the gene-sets spatiotemporal expression enrichment analysis in this study, only risk genes derived from data-driven, hypothesis-free genome-wide association/sequencing studies or alike databases were included. The up-to-date published GWAS of Intelligence Quotient (IQ) ^58^, 10 psychiatric traits, including Autism Spectrum Disorders (ASD) ^59^, Attention Deficit Hyperactive Disorder (ADHD) ^60^, Tourette’s Syndrome (TS) ^8^, Obsessive Compulsive Disorder (OCD) ^61^, Anorexia Nervosa (ANO) ^9^, Neuroticism ^62^, Panic disorder ^6^, Major Depressive Disorder (MDD) ^3,63^, Bipolar Disorder (BIP) ^5,64^, and Schizophrenia (SZgw) ^4,65^, and 3 neurological disorders, including Alzheimer’s Disease (AD) ^10,66^, Parkinson’s Disease (PD) ^67^ and Epilepsy ^68^, as well as exome sequencing studies of ASD ^7^ and Schizophrenia (SZes) ^69^ were carefully investigated to curate unbiased genome-wide risk gene sets for each trait (Table 1). For some of those studies, multiple risk gene sets with different gene mapping strategies or criteria were selected for intra-trait comparison of their spatiotemporal expression patterns. While for overall cross-trait comparisons, the risk gene sets of different mapping strategies for the same trait were merged to form a pan risk gene set for the 15 traits. Hence, we can have head-to-head comparisons of their risk gene spatiotemporal expression patterns.

### Developmental human brain gene expression data

Complete RNA sequencing datasets were obtained from the BrainSpan project website (https://www.brainspan.org/static/download.html), encompassing transcriptomic profiles from 26 anatomically defined brain regions across 42 human postmortem donors. These donors included both males and females, spanning developmental stages from 8 post-conception weeks (PCW) to middle adulthood (approximately 40 years old) and represented diverse ethnic backgrounds. Detailed information regarding sample collection, sequencing protocols, and quality control procedures is available in the BrainSpan technical white paper (https://help.brain-map.org/display/devhumanbrain/Documentation). To remove redundancy in the expression matrix, genes with duplicated names were deduplicated by averaging their RPKM values, resulting in a final dataset containing 47,808 unique genes for downstream analysis.

### Adult human brain gene expression data

The Allen Human Brain Atlas (AHBA) provides a high-resolution transcriptional profile of the adult human brain. Microarray-based gene expression data were downloaded in full from the AHBA data portal (https://human.brain-map.org/static/download/), encompassing samples from six neurotypical adult donors, ranging in age from 24 to 57 years. Transcriptome profiling was obtained from 3,702 spatially-resolved samples that cover 231 anatomically-defined brain regions. Data preprocessing and normalization were conducted by the Allen Institute using a multistep pipeline to correct for array-specific biases, batch effects, and dissection-related variability. For downstream analysis, the maximum expression value across multiple probes was selected for each of the 20,737 unique RefSeq genes per sample. To generate an averaged transcriptomic map across individuals, all 3,702 sample-level expression profiles were pooled, and mean expression values for each gene were calculated across replicated brain regions using R. Expression enrichment of risk gene sets within each brain region was then assessed, and regions were manually matched to ENIGMA-defined anatomical areas to enable cross-dataset correlation analyses.

### Single-cell transcriptome data

Both developmental and adult human brain single-cell transcriptomic datasets were used to assess the expression enrichment of risk gene sets across distinct brain cell types. Processed developmental single-cell RNA-seq data (in TPM units), merged from PEC (Developmental), Darmanis et al. (2015), and Lake et al. (2016), and adult single-cell RNA-seq data (in UMI counts), merged from PEC (Adult) and Lake et al. (2018), were obtained from the PsychENCODE Consortium portal (http://resource.psychencode.org/#).

The developmental dataset includes TPM values for 15,086 genes across 4,249 single cells encompassing 35 annotated cell types, derived from both fetal (5 to 20 PCW) and adult neocortical brain tissues. For downstream analysis, gene expression profiles were normalized at the single-cell level and subsequently averaged within each cell type, resulting in a matrix of 15,086 gene expression values across 35 cell types.

The adult dataset comprises UMI counts for 17,176 genes across 27,412 individual brain cells. For the adult dataset, raw UMI counts were transformed to counts per million (CPM) using the R package edgeR (v3.43.8). CPM values were then averaged within each annotated cell type to generate cell-type-specific expression profiles. To reduce skewness and approximate normality of gene expression distributions, a log2(x + 1) transformation was applied. These processed data were subsequently used for statistical testing of gene set expression enrichment across brain cell types.

### Risk gene sets expression enrichment analysis

The Mann–Whitney U test, a non-parametric statistical method commonly employed in gene set enrichment analysis, was used to evaluate the expression enrichment of risk gene sets across the transcriptomes. Briefly, RPKM values from the deduplicated expression matrix were ranked within each sample, with higher ranks indicating relative expression enrichment. For each transcriptome, genes from the risk gene set were mapped to the ranked list and treated as the risk group, while the remaining genes served as the non-risk control group. To quantify the degree of enrichment, the Rank-Biserial correlation coefficient (r), a non-parametric effect size metric ranging from −1 to 1, was calculated. This coefficient represents the probability that a randomly selected gene from the risk group has a higher expression rank than a randomly selected gene from the control group. One sided P values for each r were calculated and corrected by False Discovery Rate (FDR) across the samples for each gene set. To compare the spatiotemporal enrichment patterns of risk gene expression across different traits, sample-wise r values for each risk gene set were Z-score normalized. The normalized values were subsequently clustered using the R package “ComplexHeatmap” (v2.17.0) to visualize shared or distinct expression “hot-spots” across brain regions and developmental stages. Furthermore, to investigate the developmental dynamics of risk gene expression enrichment across the 15 traits, r values were averaged across all samples within each developmental stage. A moving average filter with a window size of 3 was then applied to smooth the data, resulting in 9 developmental stages derived from the original 11 time points in the BrainSpan dataset.

### The ENIGMA structure changes and correlation with disorder risk gene enrichment

Structural MRI alterations associated with major neuropsychiatric disorders were obtained from the ENIGMA Toolbox summary statistics (https://enigma-toolbox.readthedocs.io/en/latest/pages/04.loadsumstats/index.html). To enable cross-modal integration, anatomical correspondences between ENIGMA-defined brain regions and AHBA microarray samples were manually established based on the Allen Human Brain Atlas anatomical reference and prior published mappings. Structural measures from the left and right hemispheres in the ENIGMA dataset were averaged to generate a unified value for each region. The Lateral Ventricles were excluded due to their non-parenchymal and non-functional characteristics. A total of 38 brain regions with clear cross-atlas correspondence were retained for correlation analyses. To quantify the relationship between risk gene set expression enrichment (r) and structural alterations (d_icv), Spearman correlation analysis was performed across the 38 regions. For spatial visualization, r values across the matched brain regions were projected onto the FreeSurfer average surface (fsaverage5) brain template using Python packages enigmatoolbox.plotting and matplotlib (Python 3.12).

### Weighted gene co-expression network (WGCNA) based analysis

WGCNA was used to construct the gene co-expression modules based on the BrainSpan transcriptomes using the R package WGCNA (v1.72–1). Genes with low expression (total RPKM across all samples < 10) were removed. Outlier samples were identified and excluded by hierarchical clustering based on inter-sample expression distances, using a cut height of 100,000 on the sample dendrogram. For the remaining 26,598 genes in 521 qualified samples, pairwise Pearson correlation coefficients were calculated across all samples. A soft-threshold power of 7 was used to achieve approximate scale-free topology (R2 >0.85). A signed weighted gene co-expression network was constructed using the blockwiseModules function. The network dendrogram was created using average linkage hierarchical clustering of the topological overlap dissimilarity matrix (1-TOM). Gene modules were detected using the Dynamic Tree Cut algorithm with a minimum module size of 40, and each module was summarized by its module eigengene (ME), defined as the first principal component of its expression matrix.

The associations between ME expression levels and both spatial and temporal variables were evaluated using linear regression models implemented in R. Briefly, ME expression levels were treated as the dependent variable, while spatial and temporal categories were encoded as dummy variables, resulting in 11 spatial and 11 temporal dummy variables. In the regression model, Thalamus and Young Adulthood were designated as the reference levels for the two variable groups, and were absorbed into the model intercept. Thus, the intercept represented the baseline ME expression level in the thalamus during young adulthood. Predicted expression levels for other brain regions and developmental stages were obtained by summing the corresponding regression coefficients (β values) with the intercept. This approach enabled the identification of spatiotemporal expression peaks (i.e., “hot-spots”) for each module in a visually intuitive manner.

Fisher’s exact test was used to assess the enrichment of risk-associated gene sets for the 15 traits within the genes comprising each WGCNA module. To further evaluate cell type-specific expression patterns, the Mann–Whitney U test was applied to determine whether genes in each module were significantly more highly expressed in specific cell types. To facilitate downstream visualization, the resulting r values representing module-cell type associations were standardized using Z-score normalization.

### Function analysis of WGCNA module genes

Module genes identified through WGCNA were subjected to functional enrichment analysis using the KEGG and GO databases. Analyses were performed with the R package clusterProfiler (v4.9.3), using a q-value cutoff of 0.05. The minimum and maximum gene set sizes were set to 3 and 500, respectively. The top three GO terms in each category–Biological Process (BP), Cellular Component (CC), and Molecular Function (MF)–were visualized using bar plots. KEGG pathway enrichment analysis was performed for genes in each of the 16 co-expression modules associated with neuropsychiatric disorders. Enrichment was conducted using the compareCluster function from the clusterProfiler R package, with a significance threshold of q < 0.05. To further explore the relationships among enriched GO terms in the Biological Process category, REVIGO (http://revigo.irb.hr/) was used to generate semantic similarity-based visualizations.

### ISH data for exemplar module/risk genes in the marmoset brain

The ISH data for representative module/risk genes in the neonatal and 6-month-old marmoset brain were obtained from our Marmoset Gene Atlas (https://gene-atlas.brainminds.jp/). Briefly, marmoset brains were sectioned at a thickness of 28 μm, and 60 coronal sections were collected at 196 μm intervals for each probe. Of these, 20 sections spaced at 588 μm intervals were selected to comprehensively capture whole-brain spatial expression patterns of the target genes at single-cell resolution.

## Supplementary Material

Supplementary Files

This is a list of supplementary files associated with this preprint. Click to download.

• SupplementaryTables13.zip

• Supplementalmaterials0921.docx

## Figures and Tables

**Table 1:** Risk gene sets curated in this study

Risk Gene Sets	Traits/Controls	Gene Mapping Strategies	Number of Risk Genes	Study Sample Size (case)	Study Sample Size (total)	Reference
IQ_GWAS_2018NatGen	Positive Control (early)	MAGMA	507	269,867	269,867	Savage et al., 2018
IQ_GWAS_2018NatGen_ExNS	Positive Control (early)	ExNS	113	269,867	269,867	Savage et al., 2018
SZ_GWAS_2022Nature_eQTL_Fetal	Positive Control (early)	eQTL_SMR_Fetal Brain	21	76,755	320,404	Trubetskoy et al., 2022
SZ_GWAS_2022Nature_eQTL_PEC.adult	Positive Control (late)	eQTL_SMR_PEC Adult Brain	71	76,755	320,404	Trubetskoy et al., 2022
BIP_GWAS_2021NatGen_eQTL_PEC.adult	Positive Control (late)	eQTL_PEC Adult Brain	32	41,917	413,466	Mullins et al., 2021
SZ_High_Confi_Risk_PEC.adult	Positive Control (late)	PEC Regulatory Networks	301	558	1,866	Wang et al., 2018
SZ_Risk_PEC.adult	Positive Control (late)	PEC Regulatory Networks	908	558	1,866	Wang et al., 2018
Random_50	Negative Control	Randomly Selected	50	-	-	-
Random_100	Negative Control	Randomly Selected	100	-	-	-
Random_1000	Negative Control	Randomly Selected	1000	-	-	-
ASD_ES_2020Cell	ASD	Exome Sequencing	102	11,986	35,584	Satterstrom et al., 2020
ASD_ES_2020Cell_Predominant	ASD (Predominant)	Exome Sequencing	53	11,986	35,584	Satterstrom et al., 2020
ASD_ES_2020Cell_NDD	ASD (NDD)	Exome Sequencing	49	11,986	35,584	Satterstrom et al., 2020
ASD_GWAS_2019_Nat_Genet	ASD	MAGMA	25	18,381	46,350	Grove et al., 2019
ASD_SFARI_1	ASD	SFARI “High Confidence”	233	-	-	https://gene.sfari.org/
TS_GWAS_2019AJP	TS	Positional	187	4,819	14,307	Yu et al., 2019
Neuroticism_GWAS_2018NatGen	Neuroticism	FUMA/MAGMA	599	449,484	449,484	Nagel et al., 2018
ADHD_GWAS_2019NatGen	ADHD	Positional	26	19,099	53,293	Demontis et al., 2019
OCD_GWAS_2018MP	OCD	Positional	66	2,688	9,725	IOCDF-GC et al., 2018
ANO_GWAS_2019NatGen	ANO	MAGMA	76	16,992	72,517	Watson et al., 2019
PanicDisorder_GWAS_2019MP	Panic disorder	MAGMA	41	2,248	10,240	Forstner et al., 2021
BIP_GWAS_2019NatGen	BIP	MAGMA	153	20,352	31,358	Stahl et al., 2019
BIP_GWAS_2021NatGen	BIP	MAGMA	161	41,917	371,549	Mullins et al., 2021
MDD_GWAS_2019NatNeu	MDD	MAGMA	269	246,363	807,553	Howard et al., 2019
MDD_GWAS_2024NatGen	MDD	Prioritised (FUMA, MAGMA, Hi-C,TWAS)	964	88,316	991,073	Meng et al., 2024
SZ_ES_2022Nature	SZes	Exome Sequencing	32	24,248	121,570	Singh et al., 2022
SZ_GWAS_2018NatGen_CLZ	SZgw	Positional	475	11,260	35,802	Pardinas et al., 2018
SZ_GWAS_2018NatGen_PGC	SZgw	Positional	348	11,260	35,802	Pardinas et al., 2018
SZ_GWAS_2022Nature_Ext	SZgw	Extended GWAS	638	76,755	320,404	Trubetskoy et al., 2022
SZ_GWAS_2022Nature_Prot	SZgw	Portein Coding	437	76,755	320,404	Trubetskoy et al., 2022
SZ_GWAS_2022Nature_Prio	SZgw	Prioritized	120	76,755	320,404	Trubetskoy et al., 2022
SZ_GWAS_2022Nature_FM	SZgw	FINEMAP	70	76,755	320,404	Trubetskoy et al., 2022
Epilepsy_GWAS_2018NatCom	Epilepsy	FUMA	146	15,212	44,889	Epilepsy Consortium, 2018
Epilepsy_Monogenic_2018NatCom	Epilepsy	Selected Monogenic	102	15,212	44,889	Epilepsy Consortium, 2018
Epilepsy_GWAS_2018NatCom_prio	Epilepsy	Prioritized	38	15,212	44,889	Epilepsy Consortium, 2018
AD_GWAS_2021NatGen	AD	FUMA	978	90,338	226,563	Wightman et al., 2021
AD_GWAS_2019NatGen	AD	Positional/eQTL	192	21,982	63,926	Kunkle et al., 2019
PD_GWAS_2019LancetNeu	PD	Positional	97	37.7K	18.6K	Nalls et al., 2019
PD_GWAS_2019LancetNeu_QTL	PD	QTL Nominated	62	37.7K	18.6K	Nalls et al., 2019

IQ, Intelligence Quotient; ASD, Autism Spectrum Disorders; NDD, Neurodevelopmental Disorders; ADHD, Attention Deficit Hyperactive Disorder; TS, Tourette’s Syndrome; OCD, Obsessive Compulsive Disorder; ANO, Anorexia Nervosa; MDD, Major Depressive Disorder; BIP, Bipolar Disorder; SZ, Schizophrenia; AD, Alzheimer’s Disease; PD, Parkinson’s Disease; ExNS, Exonic non-synonymous variants; MAGMA, Multi-marker Analysis of GenoMic Annotation; FUMA, Functional Mapping and Annotation; eQTL, expression Quantitative Trait Loci; SMR, Summary-based Mendelian Randomization; PEC, PsychEnCode; GWAS, Genome Wide Association Studies; TWAS, Transcriptome Wide Association Studies.

## Data Availability

The authors declare that the data supporting the findings of this study are available within the paper and its supplementary materials.
